# Supplementary material for: Identification of photoperiod-induced specific miRNAs in the adrenal glands of Sunite sheep (Ovis aries)
Source: Front Vet Sci. 2022 Jul 22;9:888207. doi: 10.3389/fvets.2022.888207 (PMC9354845; doi:10.3389/fvets.2022.888207)
Supplement: Supplementary file 1 [file Data_Sheet_1.ZIP › Supplementary file/miRDeep2 file.zip!/miRDeep2 file/Novel_Structure/SP42A2/Novel_555.pdf]

Provisional ID	:	NC_019464.2_17699
Score total	:	1825.3
Score for star read(s)	:	3.9
Score for read counts	:	1817.7
Score for mfe	:	2.2
Score for randfold	:	1.6
Score for cons. seed	:	
Total read count	:	3577
Mature read count	:	3575
Loop read count	:	0
Star read count	:	2

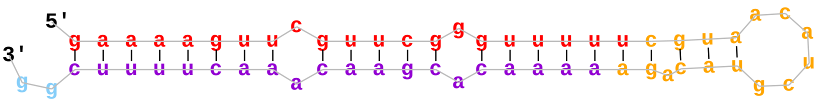

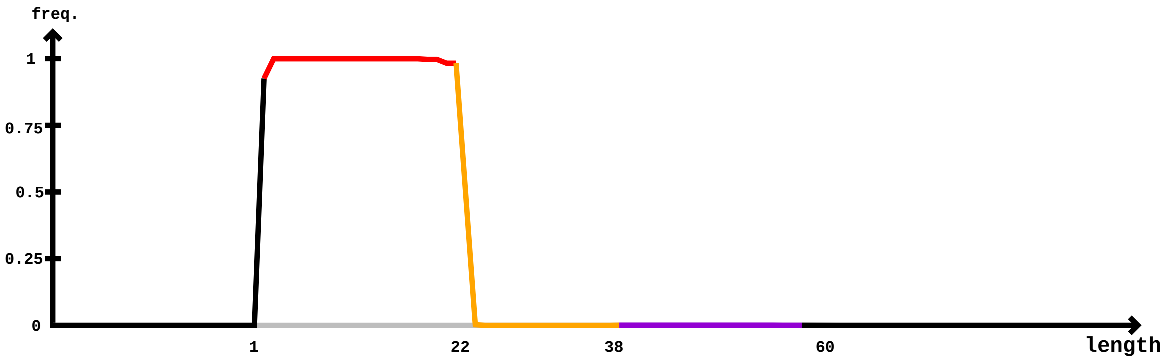

Mature Star

```
5'- auuaucauauuggguuggcugaaaaguucguucggguuuuucguaacaucguacagaaaaacacgaacaaacuuuucggccaacccaguauuucauucaucugcguuuucc  -3'  obs
    auuaucauauuggguuggcugaaaaguucguucggguuuuucguaacaucguacagaaaaacacgaacaaacuuuucggccaacccaguauuucauucaucugcguuuucc       exp
    ......((((((((((((((((((((((.(((((.((((((((((......))).))))))).))))).))))))))))))))))))))))....................  reads  mm  sample
    ....................gaaaaguucguucgggu..........................................................................  8      0   seq
    ....................gaaaaguucguucggguuu........................................................................  49     0   seq
    ....................gaaaaguucguucggguuuuu......................................................................  3253   0   seq
    ....................Aaaaaguucguucggguuuuuc.....................................................................  1      1   seq
    .....................aaaaguucguucggguuu........................................................................  2      0   seq
    .....................aaaaguucguucggguuuuuC.....................................................................  20     1   seq
    .....................aaaaguucguucggguuuuu......................................................................  146    0   seq
    .....................aaaaguucguucggguuuuuc.....................................................................  88     0   seq
    .....................aaaaguucguucggguuuuucU....................................................................  7      1   seq
    .....................aaaaguucguuUggguuuuucg....................................................................  1      1   seq
    .........................................................aaaacacgaacaaacuC.....................................  1      1   seq
    .........................................................aaaacCcgaacaaacuuuuc..................................  1      1   seq
```
